# Tools for patient-centred family planning counselling: A scoping review

**DOI:** 10.7189/jogh.14.04038

**Published:** 2024-02-02

**Authors:** Dominique Meekers, Aaron Elkins, Vivian Obozekhai

**Affiliations:** 1 Department of International Health and Sustainable Development, Tulane University School of Public Health and Tropical Medicine, New Orleans, Louisiana, USA; 2 DKT International, A. G. Leventis Building, Iddo House, Lagos Mainland, Lagos, Nigeria

## Abstract

**Background:**

The focus of family planning counselling is gradually shifting from the tiered-effectiveness model to patient-centred counselling. Although tools exist that aim to make family planning counselling more patient-oriented without increasing the provider’s workload, they are not widely used. This scoping review aims to address this by identifying key tools to make family planning care more patient-centred, reviewing the domains of patient-centred care they address, and identifying gaps in the evidence base.

**Methods:**

We systematically searched PubMed and SCOPUS for documents on ‘patient-centred family planning counselling or support’ published between 2013 and 2022. Eligibility criteria included discussion of: 1) strategies for providing patient-centred care; 2) interventions using a patient-centred approach; or 3) the impact of patient-centred approaches. We identified tools for patient-centred care, and mapped them against an existing framework of the main domains of patient-centred care. We reported the available evidence of the impact on those tools.

**Results:**

Our scoping review is based on 33 documents. We identified six tools for increasing the patient-centeredness of family planning counselling. None of the tools addressed all domains of patient-centred care. Evidence about the impact of these tools remains scarce. Although there is some evidence about the acceptability of the tools, key evidence gaps include the effect of the tools on quality of care and family planning outcomes.

**Conclusions:**

Family planning implementers should be aware that existing tools differ in the extent to which they address key domains of patient-centred family planning counselling. There is a need for further research on factors that may deter providers from adopting these tools. A larger evidence base is needed to permit a future systematic review to determine the effect of these tools on family planning outcomes, such as method adoption and continuation.

Until recently, family planning counselling was dominated by the tiered-effectiveness model, which was endorsed by the World Health Organization. In tiered-effectiveness counselling, contraceptive methods are discussed in order of effectiveness [1], which emphasises long-acting reversible contraceptive (LARC) methods. It is increasingly recognised that the tiered-effectiveness model is vulnerable to unconscious provider biases [2,3] and implicit pressure to use or not use certain contraceptive methods [4]. Focusing on method effectiveness may cause providers to overlook other factors that may be more important to the client, such as their personal values, relationship status, past contraceptive experiences, or preferences for specific contraceptive attributes. As a result, clients may adopt a contraceptive method that suits the provider’s preference, rather than their own, which can lead to dissatisfaction with the method and contraceptive discontinuation [4–7].

In recent years there has been growing interest in making family planning counselling more patient-centred. Patient-centred care, also known as client-centred care, is the provision of care that is unique and targeted to the individual’s circumstances, including the patient’s needs, preferences, and values [3,8,9]. For family planning, patient-centred counselling involves understanding the woman’s fertility goals, contraceptive needs and preferences, education on contraceptive methods, autonomy, and encouraging open dialogue [9,10]. However, providing extensive patient-centred counselling can be time-consuming, making it difficult for providers to implement [11,12]. Several family planning job aids and tools have been developed to make the provider visit more patient-centred without unnecessarily increasing the provider’s workload, but to date none of them are widely used. Their adoption may be hampered by the fact that implementers are not familiar with the range of available tools, are unclear which aspects of patient-centred care the tools facilitate, or unsure whether there is evidence of their effectiveness. More specifically, the following knowledge gaps stand out. First, while a number of documents describe a specific tool, such as a digital application or decision-making aid, to facilitate patient-centred counselling, there is no single document that provides an overview of the main tools that are currently available. Hence, unless programme implementers review a significant body of literature, it will be difficult for them to know the range of tools that are available to them. Second, given that there is no universally agreed upon definition of patient-centred family planning counselling, there is a need for clearer information about what each tool does to help make the counselling more patient-centred. McCormack’s seminal framework [13] identified six distinct domains of patient-centred care, including fostering the provider-client relationship, a reciprocal exchange of information between clients and providers, the need for providers to recognise and respond to their patients’ emotions, managing uncertainties the client may experience, making care decisions, and facilitating patient self-management. As yet, there is lack of literature that clarifies to what extent existing tools and job aids for patient-centred family planning counselling help address each of these domains. Third, there is a need for solid evidence about the extent to which various tools are deemed acceptable by both providers and patients, and about the extent to which use of these tools improves the perceived quality of contraceptive counselling, increases contraceptive knowledge, facilitates contraceptive decision-making, and increases contraceptive prevalence and continuation rates.

We conducted a scoping review to systematically address these knowledge gaps. The objective of our review is to address the following specific questions: 1) which tools (such as reproductive goals screening tools, contraceptive decision-making aids) have been used to help make family planning counselling more patient-centred, including both paper-based tools and digital applications for computers or phones?; 2) which of McCormack’s six domains of patient-centred care do the various family planning counselling tools help address? 3) what evidence is available on the acceptability of various tools by both family planning providers and patients, on the impact of such tools on patients’ reported satisfaction with contraceptive counselling, contraceptive knowledge (including awareness of potential side-effects), and family planning outcomes such as the adoption of modern contraceptive methods and continuation rates? What are the evidence gaps that should be addressed in future research?

The findings from our review will help increase awareness of the key tools used to enhance the client-centeredness of the provider-patient interaction, and will provide an enhanced understanding of how their characteristics differ. This will in turn help programme implementers decide which of these tools may be most suitable for them, which may help accelerate the adoption of such tools. An enhanced understanding of the features of the different tools may also facilitate efforts to adapt them for use in other settings, and for different modes of family planning counselling (e.g. phone-based counselling or chatbots).

## METHODS

Our scoping review is based on the framework proposed by Arksey H and O'Malley L [14] and subsequently refined by others [15–17], and follows the guidelines of the PRISMA Extension for Scoping Reviews (PRISMA-ScR) checklist [18,19]. The key elements of the study protocol, including the objectives, inclusion criteria, and methods, were discussed and agreed upon in advance. We only used an informal protocol; there is no registered or published protocol for this study. As is common with scoping reviews, the search and data extraction process were iterative [16,17,20,21]. Specifically, to develop our search strategy, we first piloted an initial list of keywords and then refined the list to ensure that our search strategy was sufficiently specific. Similarly, we piloted a draft data extraction (sometimes called ‘data charting’) table on a subset of documents, and then refined and updated it to better tailor it to the study objective and research questions.

As recommended in the guidelines for scoping reviews, our analysis is limited to basic qualitative data coding [17,22]. Specifically, we identify different tools, such as questionnaires and apps, that are being used to help make the interaction between the family planning provider and the client more patient-centred. To more clearly illustrate the differences between these tools, we classified them against the six domains of patient-centred care that are addressed in the existing McCormack framework [13]. The latter analysis aims to identify gaps (i.e. domains of patient-centred care) that are not adequately addressed by existing tools. Unlike systematic reviews, scoping reviews are not designed to synthesise or pool evidence of the effectiveness of different interventions or tools [14,20,22]. Hence, we conduct evidence mapping solely to describe what evidence is available and to identify gaps in the evidence base.

### Information sources and search strategy

Our information sources consisted of documents in the PubMed and SCOPUS databases that were published between 1 January 2013 and 31 December 2022, which ensured that our review focused on the most recent literature. Given that the shift from the World Health Organization (WHO) endorsed tiered-effectiveness family planning counselling model to the patient-centred counselling model is relatively recent, the date range covered is expected to include most of the literature on patient-centred family planning counselling. We deliberately opted to restrict the date range to completed calendar years, because doing so will provide a clear starting point for any potential follow-up reviews later on. Because client-centred family planning counselling is a new and rapidly evolving area, we deemed it important to complete the review in a timely manner. Hence, we opted to limit the review to two major databases. We included PubMed because it focuses on biomedical and health-related literature, which is arguably highly relevant for family planning. We selected SCOPUS because it is interdisciplinary and covers a much larger number of journals than other popular databases. We did not impose any restrictions of the type of document, publication status or language of the document. However, the two databases we searched focus predominantly on English-language peer-reviewed documents. We applied a comprehensive search strategy aimed at identifying the key tools or instruments that have been used to make family planning counselling more patient-centred. Consistent with other authors [23], we found that Medical Subject Heading (MeSH) terms (e.g. contraception, family planning, counselling) alone did not provide sufficient specificity for our study topic, and opted to supplement them with more focused non-MeSH terms that explicitly referred to patient-centred care or similar terms (S1 in the **Online Supplementary Document**). Our final search was conducted on 1 April 2023.

### Evidence selection

The search results were imported into the Covidence screening and data extraction tool (www.covidence.org), which automatically identified and excluded any duplicate documents. Two reviewers (DM and AE) independently screened the titles and abstracts of the remaining documents for relevance. We considered documents relevant if they discussed one or more of the following aspects of patient-centred family planning counselling: 1) a strategy or approach for providing patient-centred counselling (including theoretical approaches and approaches for assessing the clients’ needs), 2) an intervention that applied a patient-centred approach, or 3) evidence of the impact of patient-centred approaches on perceived quality of care or family planning outcomes. We did not formally restrict our review to peer-reviewed studies. However, the two databases we used are skewed heavily toward peer-reviewed literature, which resulted in a de facto exclusion of grey literature. Upon completion of the independent review for relevance by the two reviewers, we compared how the reviewers had classified the documents. If the two reviewers disagreed about the relevance of a document, or if either reviewer was uncertain about its relevance, the document was discussed to achieve consensus. A small number of documents for which we did not reach consensus were retained for full document review.

During the full document review, we excluded additional duplicate documents (e.g. conference abstracts and grey literature already described in an included published document), and documents that were not relevant or lacked sufficient detail related to our objectives. Consistent with other reviews, we excluded documents that did not address patient-centred approaches, but simply recommended implementing such approaches in the future [23]. Once again, the full document review was conducted independently by the two reviewers, after which any differences were resolved by consensus.

### Data extraction/charting

We used an Excel data template to extract (chart) relevant data. One reviewer collected data from each report, which was subsequently checked by the second reviewer. We extracted the following data about the study characteristics: author, document title, year of publication, region (USA/Europe, Africa, Asia, Latin America), document type (theoretical/conceptual paper, systematic review, methodological paper, impact evaluation, etc.). For papers that discussed the implementation of a patient-centred family planning intervention, we extracted the type of study population (e.g. family planning clients or providers), the delivery mode of the intervention (face-to-face, SMS, phone (voice or IVR), phone app, web-based, or other); name or description of any patient-centred tools or aids used) and key findings or lessons learned. For the key findings, we extracted the effect measures as reported in the document (e.g. odds ratios, percentage differences). Since the objective of the scoping review is not to synthesise the evidence, but merely to map what evidence exists (and what the evidence gaps are) no bias or quality assessment was conducted [14,20,24].

## RESULTS

### Search results

**Figure 1:**
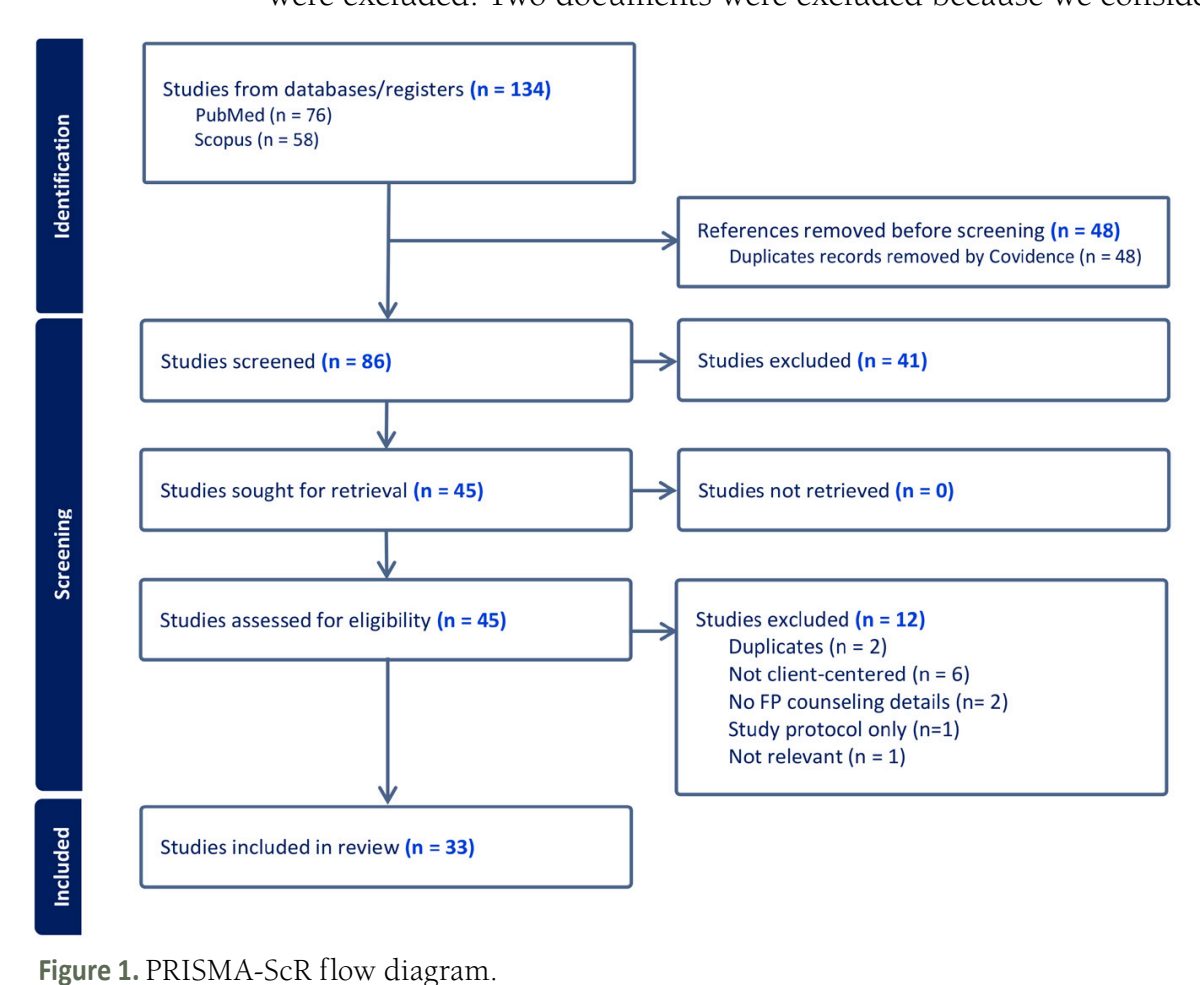
PRISMA-ScR flow diagram.

Our initial search yielded 134 results, including 76 references from PubMed and 58 from SCOPUS. The Covidence software identified and removed 48 duplicate records (**Figure 1**). Screening of the titles and abstracts of the remaining 86 unique documents resulted in the exclusion of 41 references. The remaining 45 documents were retrieved for full-text review. During the full-text review, an additional 12 documents were excluded. Two documents were excluded because we considered them to be duplicates. One of these documents – the only grey literature document – was excluded because the findings were subsequently published in peer-reviewed documents included in the review. Another document was excluded because it was a published summary of a larger article already included. We excluded six documents that did not discuss patient-centered approaches, and only recommended using such approaches in the future. Other exclusion reasons were that the study did not elaborate on the family planning counselling approach (n = 2) or only included a study protocol (n = 1). One study was deemed irrelevant because it only addressed provider perceptions about the quality of their training. Hence, 33 documents were retained for our review. The data we extracted from these documents are available on the Harvard Dataverse repository.

### Characteristics of the included literature

The 33 full-text documents retained for this scoping review included three theoretical and/or conceptual articles [1,3,6] and three systematic reviews [7,23,25]. Eight studies focused on women’s experiences with family planning counselling and counselling preferences [2,4,9,26–30], one study discussed the process of contraceptive decision-making [5], and five studies focused on measurement of patient-centred family planning care [10,31–34]. Only 13 studies discussed a tool for patient-centred family planning care. Of these, four studies discussed a reproductive goal screening tool [8,12,35,36], six a contraceptive decision-making tool [11,37–41], and three a broader counselling programme or curriculum [42–44]. Most of the studies focus on the USA (n = 20), while only four studies were done in Africa [2,26,29,40], one in the Middle East [42], one in Asia [30] and one in Latin America [28].

### Tools for increasing the patient-centredness of family planning counselling

Our review of the literature identified several tools for patient-centred family planning counselling (**Table 1**).

**Table 1:** Tools to promote patient-centred family planning counselling, by author

	Patient-Centred Care Tool						
	**One Key Question**	**Family planning quotient/reproductive life index**	**Smart Choices**	**My Birth Control**	**MyPath**	**iMacc**	**Other tools**
Baldwin et al., 2018 [12]	√⃰	√⃰					
Brandi and Fuentes, 2020 [1]	√√†						
Callegari et al., 2021 [37]				√⃰	√⃰		
Dehlendorf, Fitzpatrick, et al., 2019 [38]				√⃰			
Dehlendorf, Reed, et al., 2019 [39]				√⃰			
Dev et al., 2019 [40]						√⃰	
Donnelly et al., 2014 [41]							√⃰
Gawron et al., 2022 [8]	√⃰	√√†			√√†		
Kamhawi et al., 2013 [42]							√⃰
Koo et al., 2017 [11]			√⃰				√√†
Madrigal et al., 2019 [35]	√√†	√⃰		√√†			√√†
Morse et al., 2017 [6]	√√†						√√†
Stulberg et al., 2019 [36]	√⃰						

*√tool was applied or tested in the study.

†√√ tool was mentioned/discussed in the study.

#### One Key Question (OKQ)

Several studies used One Key Question [8,12,36]. OKQ is a screening tool that aims to: 1) help determine a patient's preferences about a future pregnancy; 2) facilitate a subsequent discussion about the patients' reproductive goals; and 3) encourage the provider to offer patient-specific contraceptive counselling. The OKQ tool was originally designed by the Oregon Foundation for Reproductive Health [45]. It is currently licensed by Power to Decide (www.powertodecide.org), which offers certification training. To use OKQ, providers are required to ask clients ‘Would you like to become pregnant in the next year? (yes; no; unsure; I am OK either way)’, which must be followed by comprehensive patient-centred counselling that is consistent with the client's response. All clients are offered preconception counselling (including screening for potential high-risk pregnancies, advice to reduce alcohol or tobacco use, etc.), and informed about the benefits of adequate child spacing and about contraceptive options. For clients who do not wish to become pregnant, information is provided about effective contraceptive methods, their correct use, and what to do in case of accidental incorrect use. Clients who are unsure about their pregnancy intentions or are OK either way, are offered both contraceptive and preconception care, tailored to their specific goals [45]. Given that there is flexibility in how the provider responds, use of OKQ does not necessarily result in patient-centred counselling [8].

#### Family Planning Quotient and Reproductive Life Index (FPQ/RepLI)

Like the OKQ, the FPQ/RepLI tool was designed to facilitate discussions and decision-making about reproductive life goals and family planning (FPQ) [12,35]. The FPQ/RepLI is described as a patient-centred tool that is intended to be incorporated into the patient’s electronic medical record. The tool visually depicts a patient’s reproductive life plan, enables tracking of progress toward the patient’s reproductive goals, and helps the provider discuss the patient’s needs and options. The FPQ/RepLI tool is completed before the patient sees the provider. To collect the necessary information for the FPQ/RepLI tool, a health educator first speaks with the patient about her sexual, gynaecologic and obstetric history and completes the tool.

FPQ/RepLI is comprised of four main components. The first component is a graph that visualises the Family Planning Quotient, which is the ratio of the number of children the patient already has, including both biological and non-biological children, over the desired number of children. When the patient’s FPQ is below one, she has not yet achieved her reproductive goals; when the FPQ equals one, her reproductive goal has been met; and when it is larger than one, she has already exceeded her desired number of children. The second part of the tool is a decision-making tree for selecting the types of contraceptives the patient should be counselled on. For women who have met or exceeded their reproductive goal, counselling focuses on reversible or permanent long-term contraceptive methods. For women, when who have not yet met their reproductive goal, the One Key Question is used to determine the type of contraceptive counselling. Women who wish to get pregnant in the next year are counselled on short-acting contraceptives methods. Women who wish to get pregnant later are counselled on both short-acting contraceptives and long-acting reversible methods. The third component of the tool is the Reproductive Life Index (RepLI), which tracks annual progress in the FPQ, pregnancy outcomes (including unplanned births), as well as other relevant outcomes (e.g. adopted children and stepchildren). Finally, the fourth part consists of a table that tracks annual changes in the type of contraceptive method used [35]. A unique feature of the FPQ/RepLI tool is that it provides a longitudinal, graphical view of the patient’s progress toward the stated reproductive goal. As such, it takes into account that a woman’s reproductive goals can be fluid and change over time.

#### Smart Choices

Smart Choices is a computer-based tool that aims to improve contraceptive counselling aid by making the counselling session more comprehensive, better tailored to the patient’s need and preferences, and by increasing the patient’s ability to have an active role in contraceptive decision-making [11]. A detailed description of the tool is provided in Wilson EK, Krieger KE, Koo HP, Minnis AM and Treiman K [46]. The Smart Choices tool is designed to be downloaded and used by a wide range of clinics, provided that they have a computer and printer. The tool is then used by patients while they wait to meet with their provider. The first component of the tool is a questionnaire that asks the patient about childbearing plans and intentions, including about things the patient may desire before having a baby (e.g. complete their education). This component also collects information about the patient’s contraceptive experience (including method satisfaction), partner influences on pregnancy preferences and contraceptive use, menstrual problems, sexual risk behaviour; it also asks the patient about questions/concerns about contraceptives or sexual health s/he would like to discuss with the provider. A printed form with the patient’s answers helps clarify to the provider which issues the patient is concerned about, and helps streamline the counselling topics.

The second component of the tool consists of an interactive, audio-visual guide that allows the patient to get in-depth information about different methods. The tool enables the patient to select contraceptive methods with specific attributes she may prefer (STI protection; non-prescription; non-hormonal; instant sex; easy to hide; works immediately; lighter periods). Contraceptive methods with the selected attributes are organised by level of effectiveness. For each method, the tool provides a two-minute audio-visual presentation and/or detailed text about the method. Smart Choices does not recommend or encourage use of any specific method [11,46].

#### My Birth Control

My Birth Control is a tablet-based interactive family planning decision-making tool used to assess women’s contraceptive values and preferences and help them select a conceptive method that matches those preferences [38,39]. Like several other tools, My Birth Control is intended to be used before visiting a provider. The tool aims to improve use of best practices in shared contraceptive decision-making. To achieve that, the tool provides information about different contraceptive methods, inquires which contraceptive features are most important to the users, conducts a short medical history check, and then recommends contraceptive methods based on the information provided by the user. A printout of the user’s answers and the recommended methods can then be shared with the provider to inform the counselling session.

The My Birth Control tool is available at https://clinic.mybirthcontrol.org. The first part of the My Birth Control tool aims to address common questions that patients have about modern contraceptive methods, including the effectiveness of the various methods, how they are used, how often the method needs to be administered or renewed, the potential side-effects of the method, and what to do if or when the patient decides she wants to get pregnant. When the user selects ‘how well does it prevent pregnancy,’ the user sees an infographic that shows that 85 out of 100 women will get pregnant during the first year of not using a contraceptive method. The user can then select a contraceptive method from a list of modern methods (arranged from most to least effective) to see how many unintended pregnancies are expected for that method. Next, the tool invites the patient to click on the icon presenting a modern method to get a short description of how it is used. Similarly, the patient can select a method to see a graph that indicates how often it needs to be used. The section of the tool that addresses potential side-effects first gives short descriptions of common side-effects that users may find positive, side-effects that one may find annoying, and side-effects that one should not worry about. The user can then select a modern method to see the specific side-effects associated with that method.

The second part of My Birth Control gathers information about when the patient thinks she may want to get pregnant, if at all, and her preferences for specific method attributes. Specially, the questions gather detailed information about the client’s preferences regarding method effectiveness, convenience of use, and the way the method is administered. The patient is also asked about her level of tolerance for several specific side-effects, including spotting/irregular bleeding, not having a period, heavier periods or cramping, and weight gain. Similarly, clients are asked how they feel about various potential method benefits (decreased acne, not having a period, decreased cramping, less heavy periods). Clients are also asked which modern method they have already used, and whether they liked the method. After inquiring about possible contraindications (high blood pressure, smoking, etc.), My Birth Control will recommend options that match the user’s preferences. The tool first shows contraceptive methods that match the preferred effectiveness, then methods that match the preferred mode of administration and frequency of use, and finally, methods that match the client’s preferences regarding potential side-effects and benefits. It also lists the methods that are not a good fit for the client’s preferences, recognising that the patient may still decide to use them.

#### MyPath

MyPath is a web-based family planning decision-support tool designed to increase reproductive health counselling and services during primary care visits, optimise the patient’s health prior to pregnancy, and increase support for family planning decision making [8,37]. Detailed information about MyPath is available at https://info.mypathtool.org/. The tool is designed to be used before visiting a primary care provider. MyPath uses a broad patient-facing approach, with a strong focus on reproductive autonomy. To achieve this, MyPath enables women to more easily communicate their reproductive goals and preferences, strengthens their self-efficacy by informing them about their contraceptive options, and improves the provider-patient relationship by encouraging patient-centered communication.

Key components of the tool include sections that: 1) solicit information about the client’s feelings and preferences regarding pregnancy and childbearing; 2) provide information about the menstrual cycle and fertility; 3) provide information about pre-pregnancy health; and 4) help identify a suitable birth control method, using the previously discussed My Birth Control tool [37]. The first section includes a question that asks the patient to articulate her reproductive preference, recognising that women may have ambivalent feelings about pregnancy (e.g. women who wish to avoid pregnancy are not necessarily unhappy if they do become pregnant). The second section clarifies when during the menstrual cycle women are most likely to conceive, and addresses misconceptions about pregnancy risk. The third section provides information about the effect of both physical and mental health on pregnancy, and aims to stimulate provider-patient discussions about actions that can improve pre-pregnancy health (including life style changes, maintaining a healthy weight, taking folic acid, etc.). Finally, the fourth section uses the My Birth Control tool to: 1) educate the patient about various aspects of different contraceptive methods (including ease of use, potential side-effects, return to fertility, etc.); 2) get information about the client’s preferences regarding these attributes; and 3) help her select an appropriate contraceptive method that is consistent with those preferences.

#### Interactive Mobile Application for Contraceptive Choice (iMACC)

iMACC is an interactive, patient-faced family planning decision-making app for mobile phones [40]. Use of the iMACC phone app is designed to streamline family planning counselling and help women make informed, personal, contraceptive choices. It was specifically designed for post-partum women, as this group may have unique preferences with respect to the features of their contraceptive method. For example, they may prefer methods that allow a quick return to fertility or methods that can be used safely while breastfeeding. iMACC is intended to be self-administered while clients wait to visit their health provider. Unlike many other tools, clients can use iMACC independently (i.e. without provider involvement) if they so desire, and the tool can help them select a contraceptive method that suits their needs and preferences by themselves. For women who prefer more provider input, the tool can help them determine which questions to ask the provider during counselling, which in turn helps streamline the counselling session.

The iMACC tool combines text and images and includes 14 health history questions and 48 queries to access individual preferences, preferences and concerns about family planning [40,47]. The health history section covers topics such as pregnancy outcomes, breastfeeding, high blood pressure, chronic headaches, cigarette smoking, etc. Family planning preferences include questions not only about the desired number of children and the preferred timing, but also how important it is for the patient to avoid pregnancy at this time. Contraceptive history questions identify the different contraceptive methods the patient has tried, assess whether they had a good experience with that method, and whether they would use it again in the future. Users are then asked to identify the three contraceptive attributes that are most important to them (effectiveness, convenience of use, concealability, reduced menstrual flow, side-effects, duration, and cost of the method). The tool also enquires about the partner’s attitude toward family planning, including whether there are any methods the partner would not feel comfortable using. This is followed by a series of detailed questions about the user’s preferences, such method convenience, concealability, menstrual flow preferences, side effects, cost, the frequency of method administration, and the time it takes to return to fertility. The tool then provides information on six modern methods, and list methods that are consistent with each category of attribute preferences (e.g. methods with the desired effectiveness, methods that avoid undesired side effects, etc.).

#### Other tools

A few studies discussed alternative tools, but lacked detailed information. For example, Donnelly KZ, Foster TC [41] intend to develop a new contraceptive decision support tool based on the Option Grid model. Other tools briefly mentioned include the Reproductive Health Self-Assessment Tool (RH-SAT), Bedsider, My Method, Method Match, and Best Method for Me [6,11]. Some authors did not use a specific tool, but relied on a broader package of tools, such as the WHO’s medical eligibility wheel, service provider and patient cue cards, and informational posters [42], or described broad training curricula [43,44].

### Mapping the tools against the domains of patient-centred care

As shown above, there is considerable variation in the content covered by the different tools that are being used to stimulate patient-centred family planning care. These content differences imply that the tools do not necessarily focus on all domains of patient-centred care. To facilitate comparison, we classified the tools against the six domains of patient-centred care (**Table 2**) that are addressed in the McCormack framework [13]: fostering the relationship between provider and client; reciprocal exchange of information; recognising and responding to patient emotions; managing uncertainty; making decisions; and enabling patient self-management.

**Table 2:** Main domains of patient-centred family planning care explicitly addressed by different tools

	Patient-Centred Care Domains					
	**Building provider-patient relationship**	**Exchanging information**	**Addressing patient emotions**	**Managing uncertainty**	**Making decisions**	**Enabling self-management**
One key question (OKQ)	*	Tool collects client’s desire to get pregnant in the next year.	*	**†**Provider provides both contraceptive and preconception care for clients who are unsure about their pregnancy intentions.	*	**†**Provider explains what do to in case of incorrect method use.
		**†**Provider counsels on preconception health, benefits of child spacing and contraceptive options.				
Family planning quotient and Reproductive Life Index (FPQ/RepLI)	Tool is part of the patient’s record, which provides continuity across multiple provider visits.	Tool tracks client’s reproductive goal relative to actual family size (incl. non-biological children) over time.	*	Tool uses a longitudinal approach, which allows reproductive goals to be fluid.	Tool shows progress toward long-term reproductive goal. Results determine whether the provider counsels on short-acting, long-acting, or permanent methods.	*
		Tool/health educator collects sexual, gynaecologic, and obstetric history.				
		*Provider counsels on tools that match reproductive goals/intentions.				
Smart Choices	Tool asks patient about questions she wants to discuss with the provider.	Tool collects patient info about childbearing plans and intentions, contraceptive history and experience, partner attitudes, sexual risk behaviour.	Tool inquires about partner influences, past experiences with contraceptives.	*	Tool identifies contraceptive methods that fit client’s preferences but does not recommend a specific method.	*
	Provider addresses questions/concerns patient listed on the tool printout.	Tool provides in-depth audio-visual information about the range of contraceptive methods.				
		Tool collects patient preferences regarding contraceptive attributes and provides detailed information about methods that match those preferences.	Tool inquires about client’s concerns about contraceptives or sexual health (open-ended).			
My Birth Control	Tool invites questions patient wants to discuss with the provider (open-ended).	Tool addresses common questions about modern contraceptive use, and detailed information about specific methods.	Tool inquires about past experiences with contraceptives.	*	Tool recommends contraceptive methods that fit client’s preferences and past experiences.	*
	Provider addresses questions patient listed on the tool printout.	Tool collects patient info about pregnancy intentions, contraceptive history and experience, and contraindications.	Tool inquires how much patient cares about various method attributes and how she feels about different side-effects or benefits.			
		Tool collects patient preferences regarding desired and undesired contraceptive attributes.				
My Path	Same as My Birth Control.	Same as My Birth Control, plus:	Same as My Birth Control, plus:	Tool allows clients to express ambivalence regarding pregnancy intentions.	Same as My Birth Control.	*
		Tool provides information about menstrual cycle, pregnancy risk, and pre-pregnancy physical and mental health.	Tool assesses emotions regarding potential pregnancy.			
Interactive Mobile Application for Contraceptive Choice (iMACC)	*	Tool collects patient info about medical eligibility, fertility and family planning intentions, contraceptive history and experience.	Tool inquires about past experiences with contraceptives; partner’s attitudes/preferences.	Tool allows clients to express ambivalence regarding pregnancy intentions.	Tool identifies contraceptive methods that fit client’s preferences.	*
		Tool provides family planning info and dispels myths and misconceptions.	Tool inquires about preference for various method attributes; concerns.			

*Up to discretion of the provider.

**†**Not included in the tool itself, but part of the associated provider certification.

The results show that the reviewed tools tend to focus mostly on four of the six domains of patient-centred care. All six tools directly address the exchange of information and all but one (five tools) directly assist with decision-making. Four tools try to address patient emotions and three implicitly recognise the need to help manage uncertainty. It is noteworthy that none of the tools directly address building the provider-patient relationship. All but one of the tools leave it up to the discretion of the provider whether or not to cover self-management during the counselling.

Although building rapport between the provider and patient is recognised as a key component of patient-centred counselling, none of the tools address it directly. Three of the tools attempt to address the power imbalance between the provider and client, by explicitly inviting the user to list any questions or concerns she may have, and that she would like the provider to address. The FPQ/RepLi tool is unique because the tool helps ensure continuity of care, which is known to be important for building a trusted provider-patient relationship. Because FP/RepLi is integrated into the patient’s medical record, the tool helps provide continuity across multiple provider visits [12,35].

All six of the reviewed tools exchange information by collecting information about the patient’s pregnancy preferences and/or reproductive goals. Except for OKQ, all tools collect information about the client’s contraceptive history. Smart Choices, My Birth Control, My Path and iMACC also inquire about the client’s experience with each of the previously used methods and preference for specific method attributes [11,37,38,40]. These same tools also reciprocate the information exchange by providing the patient with detailed information about contraceptive methods, including their effectiveness, use, side-effects, etc. The OKQ and FPQ/RepLI tools do not provide the patient with information about contraceptives and leave that responsibility to the provider.

Smart Choices, My Birth Control, My Path, and iMACC all make some provisions for clients to express their emotions [11,37,38,40]. All four of these tools inquire how the patient feels about previously used contraceptive methods. My Birth Control, My Path, and iMACC also ask how the patient feels about specific method attributes, including potential side-effects. Smart Choices and iMACC also inquire about partner influences on contraceptive use (e.g. partner attitudes, preferences for specific methods). As such, the tools help the provider identify patient emotions that may affect contraceptive preferences, which then guides the counselling.

To facilitate decision-making, Smart Choice, My Birth Control, My Path and iMACC identify and/or recommend contraceptive methods that match the client’s preferences. The RPQ/RepLI tool does not identify methods that are suitable for the client, but the level of progress toward the client’s long-term reproductive goal is used by to provider to determine whether to counsel the patient on short-action, long-action, or permanent methods. The OKQ tool does not include decision-making assistance, which is left up to the discretion of the provider.

Overall, the reviewed tools do not appear to have been designed to thoroughly manage uncertainty. Three of the tools (OKQ, My Path and iMACC) do explicitly allow clients to express ambivalence toward their pregnancy intentions [37,40,45], while FPQ/RepLI recognises that clients’ reproductive goals may change over time [12,35]. While these tools help alert the provider to this uncertainty, how to manage this is typically left to the discretion of the provider. A notable exception is OKQ, as the associated certification training requires that clients who are uncertain about their pregnancy intentions should be offered broader counselling that includes both preconception health and contraceptive options [45]. None of the reviewed tools include components that enable or facilitate self-management, almost completely leaving that up to the provider. The OKQ certification does explicitly instruct providers to explain what clients should do in case of incorrect method use.

### Evidence of the impact of patient-centred counselling approaches and tools

**Table 3** maps the available evidence about the acceptability of various tools to promote patient-centred family planning, about the effectiveness of those tools for improving the provider-patient interaction and quality of care, and about the client’s contraceptive knowledge, decision-making, method adoption, and method continuation. The reader is reminded that scoping reviews do not include bias or quality assessments of the evidence, which implies that we cannot draw conclusions regarding the relatively effectiveness of the different tools. Rather, our intent is to document what has been investigated and to identify gaps in the evidence base.

**Table 3:** Available evidence on the impact of tools to promote patient-centred family planning counselling

Tool/author	Study type		Ease of use/acceptability		Effect on quality of care and provider-patient interactions		Effect on contraceptive knowledge	Effect on contraceptive decision-making, use, and continuation
OKQ								
Baldwin et al., 2018 [12]	RCT with post-test survey (clients: OKQ n = 39, FPQ n = 37; providers OKQ n = 43, OKQ n = 36).		OKQ patients were less likely than FPQ patients to find the tool helpful and use it to track reproductive health goals (51 vs. 76%, *P* = 0.02).		OKQ and FPQ clients were equally likely to find the tool helpful in communicating their reproductive goals to their provider (68 vs. 66%, *P* = 0.88).			
					OKQ providers were more likely than FPQ providers to agree the tool helped focus their counselling, but the effect was not significant (50 vs. 37%, *P* = 0.25).			
Gawron et al., 2022 [8]	Cross-sectional pre-post patient chart review (n = 41, 52).		Patient perceptions about the screening tool were not assessed, but clients were willing to complete it, and five clients voluntarily gave positive feedback.		Chart reviews show decreased documentation of a reproductive plan (22 vs. 6%, *P* = 0.02), and no change in documentation of contraceptive counselling (20 vs. 13%, *P* = 0.36) or the patient’s contraceptive method (20 vs. 37%, *P* = 0.08).			Chart reviews show no significant change in documentation current contraceptive method (20 vs. 37%, *P* = 0.08).
Stulberg et al., 2019 [36]	Cross-sectional pre-post pilot patient survey (n = 29, 34), no control group.				The percentage of clients who said their provider discussed birth control increased from 52 to 76% (*P* = 0.04); percentage who recommended LARC increased from 10 to 32% (*P* = 0.04).			
**FPQ/RepLI**								
Baldwin et al., 2018 [12]	RCT with post-test survey (clients: OKQ n = 39, FPQ n = 37; providers OKQ n = 43, OKQ n = 36).		FPQ patients were more likely than OKQ patients to find the tool helpful and use it to track reproductive health goals (76 vs. 51%, *P* = 0.02).		FPQ clients were as likely as OKQ clients to find the tool helpful in communicating their reproductive goals to their provider (68 vs. 66%, *P* = 0.88).			
					FPQ providers were less likely than OKQ providers to agree the tool helped focus their counselling, but the effect was not significant (37 vs. 50%, *P* = 0.25).			
Madrigal et al., 2019 [35]	Post-test only study with clients (n = 790) and providers (n = 66).		Completion of the FPQ/RepLI tool by a health educator took about five minutes. 92% of patients found the tool helpful and would use it to track their reproductive goals.		Most patients agreed the tool helped them think about their personal goals (94%) and helped communicate their personal goals to the provider (90%).			
			Most providers agreed that the tool was useful to facilitate discussing reproductive health (91%) and that this type of tool is needed (83%).		Most providers agreed that the tool helped them understand the patient’s reproductive plan (91%), help focus their counselling (92%), and improved the family planning counselling they provided (73%).			
**Smart Choices**								
Koo et al, 2017 [11]	Post-test only study with intervention (n = 126) and control clients (n = 214).		The average completion time was 14 min.		In multivariate analyses, intervention women rated their visit more patient-centred than controls (mean score 3.9 vs. 3.7, *P* < 0.05). Intervention women reporting discussing more sexual health topics than control women (1.2 vs. 0.9, *P* < 0.10). No effect was found on the number of childbearing-related topics that were discussed.		After controls, intervention women knew 11.1 contraceptive methods vs. 10.7 for the control group (*P* < 0.001).	After controls, intervention women were less likely than controls to adopt IUDs or implants (9 vs. 20%), and more likely to select oral contraceptives (64 vs. 54%, *P* < 0.10).
**My Birth Control**								
Dehlendorf, Fitzpatrick, et al., 2019 [38]		RCT with post-test survey of providers (n = 28) and clients (n = 758).				Intervention clients reported higher interpersonal quality of counseling (OR = 1.45 (1.03–2.05)) and greater satisfaction with side-effects information (OR = 1.61 (1.11–2.33)). The tool had no effect on patient satisfaction with how the provider helped with method choice (OR = 1.30 (0.93–1.82)).	The tool improved knowledge of several contraceptive attributes. E.g. intervention clients were more likely to know that IUDs are more effective than pills (OR = 2.65 (1.94–3.62)), that methods that cause period to stop are safe (OR = 1.86 (1.28–2.71)), and that implants to not affect fertility (OR = 1.54 (1.14–2.07)).	The tool had no effect on satisfaction with the chosen method (OR = 1.19 (0.88–1.61)), or on method continuation at seven months (OR = 0.89 (0.65–1.22)).
Dehlendorf, Reed et al., 2019 [39]		Qualitative assessment of providers (n = 15).		All providers found it acceptable and feasible to incorporate the tool in their practice. Some noted that use of the tool prior to the visit sometimes slowed clinic flow. Most providers noted the tool was acceptable to clients but could be difficult for patients not used to the technology. Use of the tool increased overall visit time by 12 min.		Nearly all providers reported the tool made contraceptive counselling more efficient and let them allocate more time to issues the patient wanted to discuss.	Providers reported the tool improved patient’s pre-counselling knowledge of contraceptive options and method attributes.	Providers said the tool helped patients be more engaged and active in contraceptive method selection.
**My Path**								
Callegari et al., 2021 [37]		Cross-sectional pre-post pilot patient survey with intervention (n = 30) and control group (n = 28).		Most clients liked the tool, found it easy to understand and felt comfortable answering the questions. Average completion time was 11 min.		Most providers agreed it made counselling more efficient and helped them discuss pregnancy goals and contraceptives. 93% of intervention clients vs. 68% of control clients reported discussing pregnancy or contraceptive needs (*P* < 0.05). Scores for self-efficacy in communicating with providers improved more among intervention than control clients (0.8 vs. 0.2, *P* < 0.05). Use of the tool did not affect clients’ rating of provider communication quality.	Scores for correct knowledge improved more among intervention than control clients (1.7 vs. 0.2, *P* < 0.01).	The tool had no significant effect on the likelihood of switching from non-prescription to prescription contraceptive methods.
				Providers did not think the tool increased their workload or hurt the clinic flow.				
**iMACC**								
Dev et al., 2019 [40]		Qualitative assessment of clients (n = 25) and providers (n = 17).		Most clients and providers reported that iMACC was easy to use and self-explanatory; clients had no issues comprehending questions and material. Average completion time was 15 min. Most providers said iMACC reduced their workload because it addresses common questions; a few worried that women’s increased knowledge about contraceptives would take longer to counsel.		Clients valued the confidentiality of the tool and felt it would allow adolescents to answer more honestly.	iMACC helped clients understand their contraceptive options and potential side-effects and dispel myths.	iMACC made clients feel empowered to make informed decisions about methods most suitable for them.
						Most providers noted the tool allowed women to ask more questions; some claimed most women had already decided on a method before meeting them.		

Several studies have investigated the acceptability of the different tools, for both providers and clients [8,11,12,35–40]. There is scattered evidence on the tools’ ease of use and the time needed to complete them. A few studies have examined whether the tools affected the patient flow or the provider’s workload. The effect of the tools on various aspects of quality of care has also been examined fairly extensively [8,11,12,35–40]. Studies of OKQ and FPQ/RepLI have examined whether these tools helped clients to think about their personal reproductive goals and to communicate those goals to their provider [12,35,37]. There is also some evidence on the extent to which various tools can help providers to focus their counselling [12,35,37,39]. Studies of My Birth Control and iMACC examined whether such tools can make the counselling more efficient by enabling providers to spend more time on issues the patient wanted to discuss and by allowing clients to ask more questions [39,40].

A number of studies have examined whether Smart Choices, My Birth Control, My Path and iMACC have helped increase knowledge of contraceptive methods and their attributes [11,37,38,40]. Studies on the effects of the tools on contraceptive outcomes remain scarce and have focused on topics such as contraceptive decision-making [40], method adoption [11], method choice [37,39], and method continuation [38]. As yet, there is insufficient evidence to assess the relative efficacy of the different tools for improving family planning outcomes.

## DISCUSSION

Our first research objective was to identify tools that are being used to help make family planning counselling more patient-centred. We identified six tools for making family planning counselling more patient-centred. Two of these tools focus on the clients’ reproductive goals, while the other four are better described as contraceptive decision-making tools. Although there has been substantial interest in shifting from tiered-effectiveness family planning counselling to patient-centred counselling during the last five years [1,3,25], providers may be reluctant to implement patient-centred counselling because it tends to be more time-consuming [25]. Hence, our findings are important to increase providers’ awareness that several tools are at their disposal to help facilitate this shift by providing tailored approaches for being more responsive to the patients’ reproductive goals, values and preferences.

To better understand how these tools operate, our second research objective was to assess which of McCormack’s six domains of patient-centred care each tool is trying to address [13,23]. We found that none of the tools fully address all domains of patient-centred care. The domains most emphasised are ‘the exchange of information’ and ‘facilitating decision-making’. All tools collect information about the client, which then guides the counselling, and four of the six tools are decision-making aids. Although ‘fostering the relationship between provider and client’ is a quintessential domain of patient-centred care, none of the tools address it directly. However, several of the tools include provisions for the clients to list any questions or concerns they would like the provider to address, which may help address the power imbalance. To some extent, the four contraceptive decision-making tools facilitate ‘recognising and responding to patient emotions’ by inquiring how the patient feels about previously used contraceptive methods, specific method attributes, and potential partner influences. As expected, the four contraceptive decision-making tools identify and/or recommend contraceptive preferences that match the client’s needs and preferences. Hence, the tools may help providers to rapidly determine the patients’ reproductive life goals and/or their contraceptive values and preferences, which would help address concerns that patient-centred family planning counselling is too time-consuming to implement [25]. Generally speaking, the tools do little to directly address the domains ‘managing uncertainty’ or ‘enabling self-management’, both of which are largely left to the discretion of the provider. The omission of the management of uncertainties is an important gap considering that patients may be ambiguous regarding their reproductive goals, for example because their goal conflicts with that of their partner, and because contraceptive experiences and preferences for contraceptive method attributes tend to change over time [5,12]. Similarly, the lack of attention to self-management is notable, given that method side-effects are an important reason for contraceptive discontinuation [48,49]. There is a need for a more widely accepted definition of patient-centred family planning counselling and its sub-domains, to ensure that future tools address all aspects of patient-centred family planning care.

Our final objective was to document what evidence is available on the acceptability of the tools to facilitate patient-centred family planning counselling and on the effect of their use on family planning outcomes. Our review shows that the evidence base on these subjects is still very small, and that much of the evidence stems from fairly small pilot studies. Provider acceptability of the tools is essential for them to be widely adopted. As noted by Baldwin MK, Overcarsh P [12], ‘Given time constraints in clinics, any job aid needs to be easy to integrate and efficient, and should provide enough information to facilitate individualized counseling’. Hence, there is a need for further investigation of potential provider concerns that may either deter providers from adopting these tools or that may lead them to subsequently cease their use. We did not identify any studies that examined provider resistance to the tools prior to their adoption. Although some studies examine whether use of the tools affected the providers’ workload or patient flow, their study designs and indicators are not comparable across tools [11,37,39,40,42]. To enable comparisons across tools, it is recommended that future studies report standardised indicators, such as the percentage of providers and percentage of patients who are satisfied with the tool, the total patient visit time (including time to interact with the tool), the time providers spend with the patient, and the total patient/clinic flow. Since patient-centred counselling is known to be more time-consuming than tiered-effectiveness counselling [25], future studies of the provider workload should distinguish between the effect of changing the counselling mode and the effect of using the tools. For example, it would be helpful to have studies that compare the average duration for patient-centred family planning counselling with and without each of the tools.

As yet, there is a dearth of evidence on the effect of contraceptive counselling tools on clients’ perceptions about the quality of care, knowledge of contraceptive methods (including side-effects), or empowerment to make informed decisions about which contraceptive method is best suited for their own needs and preferences. Further research is needed to assess whether improvements in the counselling experience generated by these tools also translate into better family planning outcomes, such as improved satisfaction with the client’s chosen contraceptive method or reduced method discontinuation. This will require impact evaluations with rigorous study designs. Although it may not be feasible to implement large-scale randomised controlled trials for each tool, studies that use a pre-post design with a comparison group and studies that use propensity score matching to create a comparison group can also help strengthen the evidence base. To help providers choose between different tools, it would be helpful for future studies to use comparable family planning outcome indicators, such as the percentage of clients who adopt a modern contraceptive method, who are satisfied with their chosen method, and who are still using their chosen method after a fixed time interval (e.g. after three, six, or 12 months).

Future research should expand testing of these tools in low-income countries and other contexts, where they may be hard to use in their current form. Several studies suggest that background characteristics, such as race, ethnicity, age, geographic location, sexual orientation, pregnancy norms, and pregnancy history, affect the acceptance and effectiveness of these family planning tools [5,31,35,40]. Hence, tailoring the content of the tools to the specific cultural context may increase their effectiveness. In numerous societies, open discussions about sexual and reproductive health are stigmatised, particularly for younger unmarried women, which can affect the interaction with the family planning provider as well as family planning adoption [50,51]. Internalised stigma, such as shame, shyness, or fear of being judged by the provider may deter clients from disclosing the information requested by the tool, or from using the tool altogether. In such context, special attention should be paid to ensure that the content of the tools is perceived as non-judgemental (e.g. by not enquiring about the patient’s marital status) and that the tool clarifies why specific information is needed. Tools should also be designed and used in a manner that ensures patient privacy and confidentiality. For example, although most tools are intended for use in the waiting area prior to meeting with the provider, there may be a lack of privacy in the waiting area. If so, use of the tool should be delayed until the patient is meeting privately with the provider.

Providers should also consider whether the mode of implementation of the tool is appropriate for the target population. For example, use of the tools may be hampered because the health facility does not have the required technology or because clients may not have the skills or digital literary to use them [39]. Because of such constraints, it is important to select tools that are appropriate for the local context, or to adapt the tools accordingly. For digital tools, this could entail adding more visual images, audio, or video to help clarify the content and increase comprehension [40].

The current tools tend be computer or smart-phone based, which further limits their use in low-income settings. However, in many low-income countries large segments of the population have a feature phone (i.e. a phone with voice and text message capabilities, but only limited internet features). Feature phones are already being used extensively to access family planning information hotlines, including both operator-assisted hotlines and IVR (Interactive Voice Response) services. Feature phones also provide access to WhatsApp-based health information services, including family planning information. Given the widespread use of feature phones to access existing family planning information and services, there are important opportunities to adapt tools for patient-centred counselling for use WhatsApp or similar services. For the time being, it is recommended that providers consider using paper-based versions of the current tools for patients with limited digital literacy. Further research is needed to investigate which mode of implementation is most suitable in different contexts and for different population subgroups.

Family planning implementers should be aware that existing tools differ in the extent to which they address the main domains of patient-centred care. A better understanding of these differences can help implementers select the tool that best addresses their clients’ needs. Future research should also try to identify which of the six domains of patient-centred family planning counselling are most salient for improving the acceptability of the tool, perceived quality of the counselling, and family planning outcomes. Ultimately, widespread use of tools for client-centred family planning counselling is unlikely to occur in absence of solid evidence of their effectiveness. There is a need to expand the evidence base to permit future systematic reviews to determine to what extent these tools are effective for improving quality of care, contraceptive method adoption and continuation.

### Limitations of the review process and evidence

Our scoping review has several limitations. Limiting the date range for our search to the last ten completed calendar years (2013–22) may have introduced a publication bias in our findings. Because the shift from the tiered-effectiveness family planning counselling model to patient-centred counselling occurred relatively recently, we are confident that this time period covers most of the literature on the subject (of the 33 retained publications, only two were published prior to 2015). However, because this is an emerging area, it is likely that additional tools will be developed in the near future, and that the body of evidence of the efficacy of the tools discussed in our review is likely to increase rapidly. Hence, practitioners who are making decisions about which tools to implement should also consider the findings from relevant studies published since our review data range.

Our scoping review was limited to two databases, which implies that we may have missed other relevant articles. Our choice of databases resulted in a de facto exclusion of grey literature and non-English publications. Although the 86 unique documents identified in our search included only one non-English publication, expanding our search to include grey literature may have included more foreign language publications. The omission of foreign language publications may have introduced a language bias in our findings, for example if such publications had a different perspective on what comprises patient-centred family planning counselling. Although our review included a small number of studies on non-Western countries, it is unclear whether that helped mitigate this potential language bias.

Most literature on patient-centred family planning counselling focuses on support with the initial method choice; few studies address counselling and support for family planning customers after they have adopted their chosen method. Tools to promote patient-centred family planning are relatively new and evidence about their effectiveness remains scarce. Because scoping reviews do not include bias or quality assessments, we are unable to draw conclusions about the relative effectiveness of the different tools. Drawing such conclusions will require a full systematic review or meta-analysis [22]. A much larger evidence base on the effects of these tools is needed to enable such analyses.

## CONCLUSIONS

Several tools and job aids exist that are designed to help providers offer patient-centred family planning counselling. However, none of the identified tools address all six domains of patient-centred care, and the specific domains that are addressed vary across tools. Understanding these differences can help providers identify a suitable tool for facilitating counselling that is consistent with their clients’ reproductive goals, contraceptive needs and preferences. Unfortunately, the evidence base on the acceptability and effectiveness of the various tools is small. A much larger evidence base is needed to permit future systematic reviews to determine to what extent these tools are effective for improving quality of care, contraceptive method adoption and continuation.

## Additional material


Online Supplementary Document
