# Supplementary material for: Tools for patient-centred family planning counselling: A scoping review
Source: J Glob Health. 2024 Feb 2;14:04038. doi: 10.7189/jogh.14.04038 (PMC10846870; doi:10.7189/jogh.14.04038)
Supplement: Online Supplementary Document [file jogh-14-04038-s001.pdf]

S1: Searches by database

PubMed

#	Keywords/Boolean operators	Nr. of hits
#1	("family planning"[Title/Abstract] OR contraception[Title/Abstract]) AND ("counseling"[Title/Abstract] OR "support"[Title/Abstract] OR "follow up"[Title/Abstract]) AND ("user-centered"[Title/Abstract] OR "client-centered"[Title/Abstract] OR "patient-centered"[Title/Abstract])	91
#2	#1 AND ("2013/01/01"[Date - Publication] : "2022/12/31"[Date - Publication])	76

SCOPUS

#	Keywords/Boolean operators	Nr. of hits
#1	(TITLE (("family planning" OR contracept) AND ("counseling" OR "support" OR "follow up") AND ("user-center" OR "client-centered" OR "patient-centered")) OR ABS (("family planning" OR contracept) AND ("counseling" OR "support" OR "follow up") AND ("user-center" OR "client-centered" OR "patient-centered"))) AND PUBYEAR > 2012 AND PUBYEAR < 2023	58
